# Bal Poshan Yojana: A Novel Approach to Facility-Based Severe Acute Malnutrition Management

**DOI:** 10.7759/cureus.28124

**Published:** 2022-08-17

**Authors:** Jimeet Soni, Faisal Sheikh, Tanveer M Umallawala, Abid Qureshi, Somen Saha, Apurva Ratnu, Manoj S Patil

**Affiliations:** 1 Public Health, Indian Institute of Public Health, Gandhinagar, IND; 2 Epidemiology and Public Health, Indian Institute of Public Health, Gandhinagar, IND; 3 Public Health, Niramay Charitable Trust, Gandhinagar, IND; 4 Research and Development, Jawaharlal Nehru Medical College, Datta Meghe Institute of Medical Sciences, Wardha, IND

**Keywords:** mortality, facility based, management, severe acute malnutrition, bal poshan yojana

## Abstract

Introduction

Severe acute malnutrition (SAM) carries severe implications for a child’s development. The survival of a child suffering from severely acute malnourishment is majorly dependent on the management of SAM, and scaling up the framework for addressing malnutrition is one of its main components. A severely malnourished child has a higher risk of mortality as compared to an ideally nourished child. Bal Poshan Yojana under the aegis of Project Tushti, a public-private partnership (PPP) model, aims at strengthening the framework of treatment for SAM children by working towards building a sustainable eco-system through engaging the government body, private practitioners and implementing bodies.

Methods

Bal Poshan Yojana is a novel approach implemented for the first time in the Devbhumi Dwarka district of Gujarat for the management of SAM under a PPP model. The private practitioners and centres were empanelled and trained on the treatment of SAM. SAM children with medical complications were screened through anthropometric measurements and appetite tests carried out by Rashtriya Bal Swasthya Karyakram (or RBSK, a program for child health) medical team. High-risk cases were referred to the nearest empanelled Bal Poshan Centre in the area. Children were treated for 14 days at the Bal Poshan Centre as per the protocol of the National Rehabilitation Centre and then discharged if the children fulfilled the criteria. The treatment included a 14-day treatment protocol and three follow-ups in an interval of 15 days each. The data was analyzed with appropriate statistical tests.

Results

Since its inception, a total of 102 severely malnourished children under five years of age have completed their treatment successfully, including three follow-ups. Around 60.79% of SAM children have been found to become normal in their nutritional status. The mean weight gain upon discharge was 0.57 kg and after three follow-ups, it was 1.051 kg.

Conclusion

Bal Poshan Yojana is a one of its kind initiative to tackle the growing burden of malnutrition among under-five age group children. The initiative has a focused approach. Strong referral and verification mechanisms ensure complete transparency and yielding of desired results.

## Introduction

Severe acute malnutrition (SAM) in under-five age group children is a major public health concern because it is associated with high mortality and other health consequences. An estimated one-third of the world’s children who are wasted live in India. SAM is defined as severe wasting (weight for height, or WFH, Z score <-3 SD, mid-upper arm circumference, or MUAC, <115 mm or <11.5 cm, and bilateral pitting edema) as per WHO reference standard [1]. Other medical complications include anorexia, fever, persistent vomiting, dehydration, hypoglycemia, severe anemia, extensive superficial infection and any other general signs. It is a life-threatening condition among under-five children that requires immediate attention and proper management to reduce mortality and promote recovery from it. As per National Family Health Survey-5 (NFHS-5) conducted in 2019-20, Gujarat has 25.1% of children under five years of age who were wasted and 10.6% had severe wasting [2]. The NFHS-5 recorded 26.1% wasted and 17.2% severely wasted under-five children in the Devbhumi Dwarka district. The situation appeared worse as the district has more severely wasted under-five children than the state’s average.

The criteria for the identification of children with severe acute malnutrition include WFH less than -3 SD, MUAC less than 11.5 cm and bilateral pitting edema. To determine the best course of treatment for a child with SAM, a medical assessment and an appetite test are needed. About 85%-90% SAM children without medical complications and who passed the appetite test require community-based management of SAM (CMAM), whereas 10%-15% of SAM children with medical complications and a failed appetite test require facility-based management of SAM (FSAM) [3].

Children with SAM have nine times higher mortality than normal children. Various initiatives are being implemented by the Government of India to address SAM across the nation, though the main approach remains in-patient care through Child Malnutrition Treatment Centres (CMTCs) and National Rehabilitation Centres (NRCs) [3-4]. The condition of SAM children often worsens because healthcare providers unknowingly use practices that are suitable for most children, but dangerous for severely malnourished children, who are highly susceptible to nosocomial infections [5-8].

Malnutrition in children is associated with substantial morbidity, mortality and disability in the short term, and impaired cognitive development, metabolic disorder, increased risk of diseases due to recurrent infection and suboptimal economic productivity in the long term. It could be improved through the effective implementation of nutrition-specific interventions addressing the factors such as inadequate nutrition and food intake, poor caregiving, poor feeding and parenting practices, burden of infectious diseases, and nutrition-sensitive factors such as inadequate caregiving resources available to children at a maternal, household, or community level, food insecurity, limited access to healthcare services, and unhygienic environment [9]. CMTCs and NRCs have been set up at the facility level to provide medical and nutritional care to SAM children under five years of age who have medical complications. However, the uptake of these services has been a challenge in India for various reasons such as challenges for parents while staying for 14 days at a CMTC, and lack of awareness regarding the treatment of SAM in the community, among others [10-11]. Distance to facilities like CMTCs and NRCs is another reason for poor usage of malnutrition treatment [12].

Project Tushti, a three-year (2019-2022) initiative in the Devbhumi Dwarka district of Gujarat focused on tackling the malnutrition burden in the district. A baseline assessment in 2020 revealed that about 50% of institutional deliveries took place in a private setting, which highlights the faith in and preference for private practitioners. Public-private partnership (PPP) is acknowledged as an effective strategy to address the global double burden of malnutrition. The PPP approach is also encouraged under Prime Minister's Overarching Scheme for Holistic Nourishment (POSHAN) Abhiyan. A transformative approach is required to engage multisectoral stakeholders together with governments, public health agencies, or non-governmental organizations, with the private sector taking a subordinate role [13].

## Materials and methods

‘Bal Poshan Yojana’, an initiative to reduce malnutrition through public-private partnership for the treatment of severe acute malnutrition in Devbhumi Dwarka, is implemented by the Indian Institute of Public Health (IIPH), Gandhinagar, in collaboration with the Health and Family Welfare Department of Devbhumi Dwarka and supported under Project Tushti by Nayara Energy. It is a pilot scheme being implemented for the first time in India with a PPP model for facility-based SAM management in the Devbhumi Dwarka district. A total of 30 beds were reserved among three empanelled centres for the management of SAM children. The objective was to engage private nursing homes to have at least one allopathy doctor and build the capacity of public and private allopathic medical practitioners for the treatment of severe acute malnutrition.

Empanelment criteria for centres/private practitioners

The empanelment of centres was based on the following selection criteria: (1) having MBBS (minimum) doctors, (2) any registered hospital with an MBBS/MD doctor working as a visiting consultant, (3) having registered nursing staff, (4) having a minimum five-bed indoor patient department facility, (4) with fire safety, (5) having anthropometric measurement apparatus, etc. The willing private practitioners or non-governmental organizations (NGOs) were empanelled by signing the three-party memorandum of understanding (MoU) between the IIPH, Gandhinagar, the willing private practitioner or NGO, and the District Health Department of Devbhumi Dwarka. The MoU was signed for one year and then renewed.

Training of empanelled practitioners

The empanelled private practitioners under Bal Poshan Centres were trained on facility SAM management guidelines by UNICEF and Ministry of Health and Family Welfare (MoHFW), Government of India (GoI), 2011.

Referral mechanism

SAM children with medical complications were screened through anthropometric measurements and an appetite test carried out by Rashtriya Bal Swasthya Karyakram (or RBSK, a program for child health) medical team. High-risk cases were referred to the nearest empanelled Bal Poshan Centre in the area. Individual Bal Poshan Centres also did the screening campaign for SAM children, although enrollments of SAM children were done after being verified by RBSK medical officer (MO).

Criteria for SAM children enrollment

**Figure 1:**
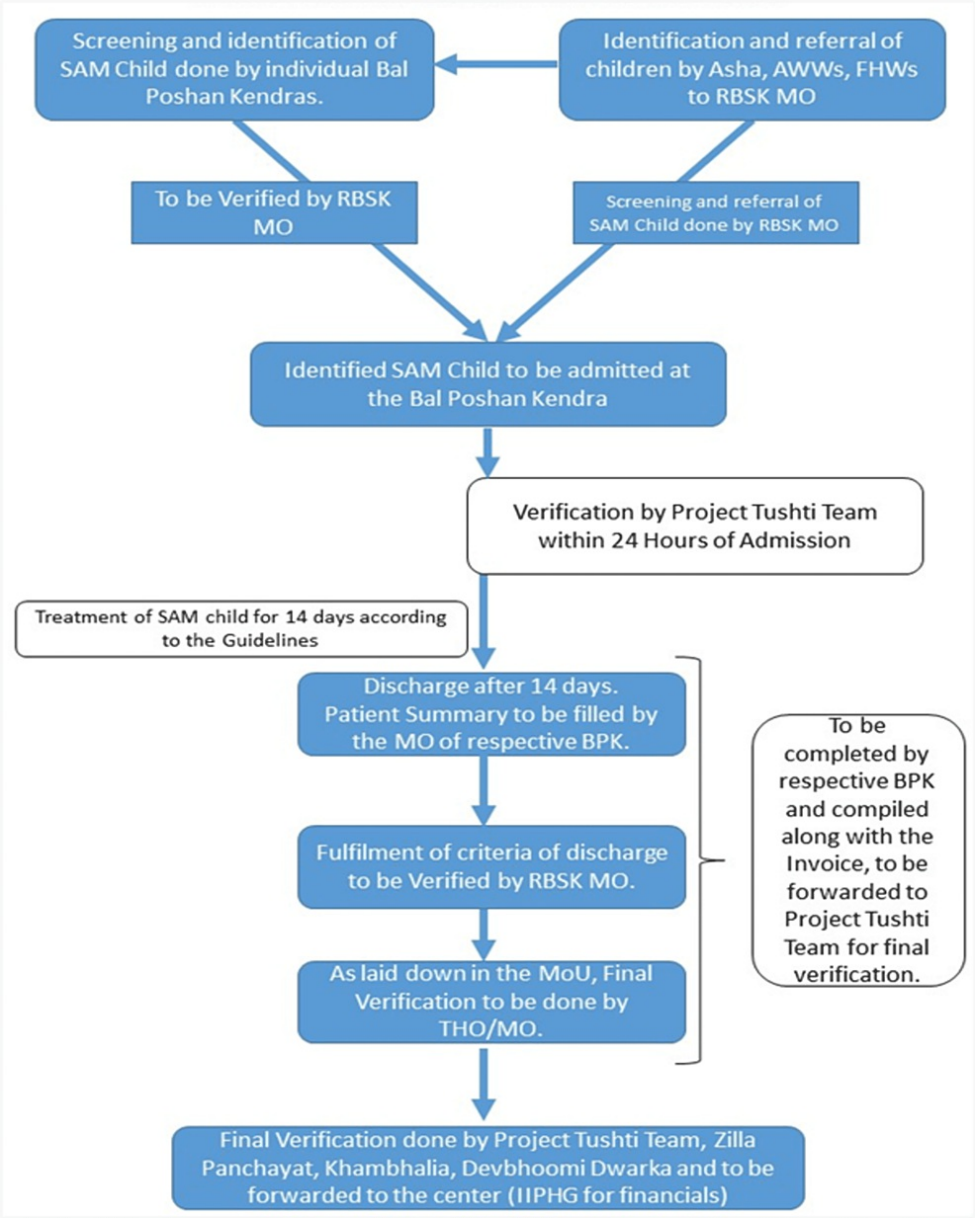
Process flowchart for Bal Poshan Yojana SAM, severe acute malnutrition; RBSK, Rashtriya Bal Swasthya Karyakram; AWW, anganwadi worker; FHW, frontline health worker; MO, medical officer; BPK, Bal Poshan Kendra (centre); MoU, memorandum of understanding; THO, taluka (town) health officer; IIPHG, Indian Institute of Public Health, Gandhinagar

The Bal Poshan Centre enrolled children of 6-59 months of age using the following criteria: MUAC <11.5 cm and/or WFH <-3 SD, appetite test fail, medical complications (anorexia, fever, persistent vomiting, dehydration, hypoglycemia, severe anemia, extensive superficial infection and any other general signs) and bilateral pitting edema. The process flow of the Bal Poshan Yojana is shown in Figure 1.

Treatment protocol

Children were treated for 14 days at the Bal Poshan Centre as per the protocol of the National Rehabilitation Centre and then discharged if the child fulfilled the criteria. Children were provided with energy-dense nutritional supplement (EDNS), Formula-75 (F-75), and Formula-100 (F-100) as per the treatment stage and criteria [14]. For the stabilization phase, a child needs around 1.5 sachets of F-75, 933 ml/day, and six sachets for a three-day course of treatment. For the transition phase, a child needs around three sachets of F-100, 1400 ml/day, and 33 sachets for an 11-day course of treatment. For the rehabilitation phase, a child requires two sachets of energy- and protein-dense food (EPDF) per day for first four weeks and one sachet for the next four weeks. The dosages are as per the Operational Guidelines on Facility-Based Management of Children With Severe Acute Malnutrition, by the MoHFW, GoI [15].

Monitoring

The treatment progress was monitored by healthcare personnel at the centre, and the same was verified by the RBSK medical officer and project team on admission, discharge and three respective follow-ups. After the treatment duration of 14 days, the RBSK team evaluated the child.

Upon successful completion of the treatment and targeted weight gain, reimbursement at the rate of 25,000 INR or 332 USD per child was provided to the empanelled centre. It includes stay and bed charges for 14 days, consultation charges, nursing charges, F-75/F-100 and EDNS, medicine charges, meals for the SAM child and the mother, the incentive for referral, transportation cost, and contingency cost. Three-fourth or 75% of incentives were paid on recovery of a child from malnutrition on discharge; the remaining 25% of incentives paid were relieved after the completion of three consecutive follow-ups at 15-day intervals. In the case of partial recovery of the child or no recovery after enrollment, 75% of incentives are to be provided. Private practitioners’ consultation workshops for the facility-based SAM management of the Devbhumi Dwarka district helped determine the compensation of 25000 INR per child under the scheme.

## Results

The pilot scheme was operational in September 2021 in the Khambhaliya block of the Devbhumi Dwarka district. Two private practitioners and one NGO partnered with the scheme. The training session for RBSK MOs, Child Development Program Officers, and AAA (the anganwadi worker, or AWW, accredited social health activist, or ASHA, and auxiliary nurse midwife, or ANM) staff was held for their capacity building to engage the beneficiaries for screening and referral of SAM children to the Bal Poshan Centre. RBSK MOs were provided with a referral form. The orientation program on screening, admission, feeding protocols, and management of facility-based severe acute malnutrition training was given to the empanelled private practitioners and their staff and RBSK medical officers of the district. The empanelled centre was provided with the ready reckoner based on operational guidelines of FSAM, charts, and summary formats.

Coverage

From September 2021 to May 2022, a total of 236 children were referred to the Bal Poshan Centres by RBSK MOs, out of which 155 (65.67%) have been enrolled and 102 (65.80%) children have already completed treatment; treatment is considered complete when a child has completed 14 days of treatment along with three consecutive follow-ups on 15 days of interval after discharge. There was a dropout of 10 (6.45%) children as the parents were not willing to come for treatment (Table 1). Each child was admitted after being cross-verified by the project team, although 81 (34.32%) children who didn't match SAM requirements at the time of admission were not enrolled.

**Table 1:** Program particulars SAM, severe acute malnutrition; RBSK MO, Rashtriya Bal Swasthya Karyakram medical officer

Particulars (for September 2021 to May 2022)	Achievement
No. of SAM-affected children referred by the RBSK MO	236
Children enrolled for treatment	155
Children who dropped out	10
Children who completed treatment (along with 3 follow-ups)	102

Program impact

**Figure 2:**
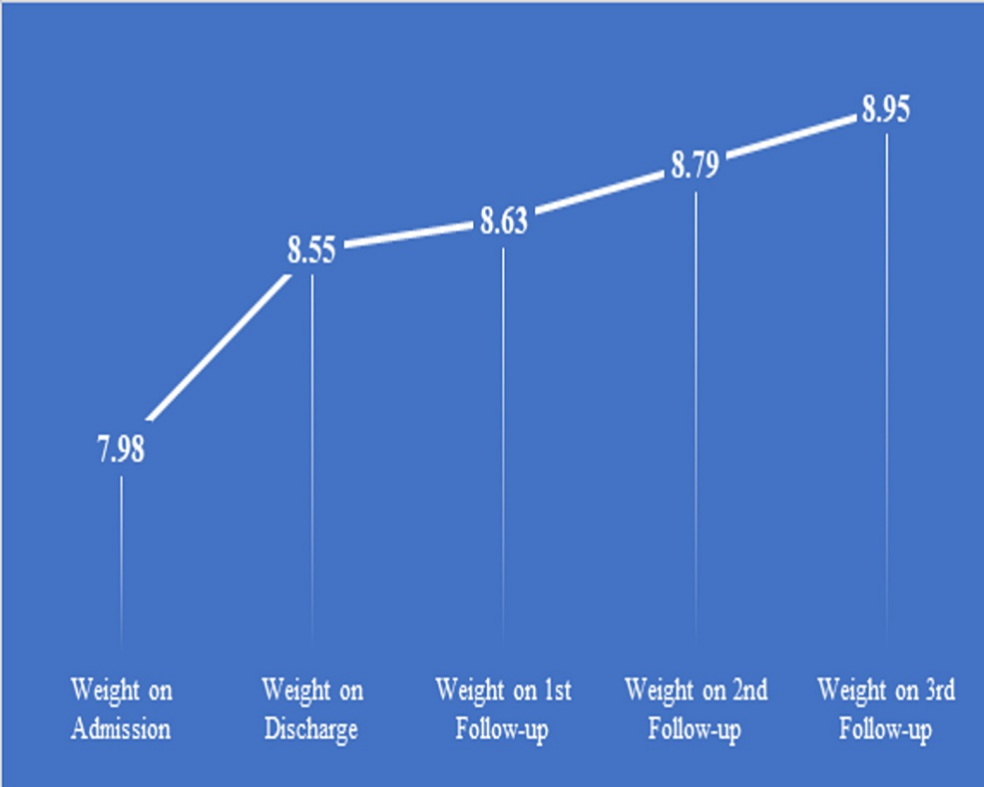
Mean weight gain in children who completed their treatment under the scheme

A total of 102 children who had completed their treatment showed significant improvement. The average weight of 102 children on admission was 7.98 kg that increased to 8.55 kg on discharge after 14 days from the Bal Poshan Centre. However, the mean weight showed significant improvement during three follow-ups at an interval of 15 days each, i.e. 8.63 kg on the first follow-up, 8.79 on the second follow-up and 9.95 on the third follow-up (Figure 2).

**Figure 3:**
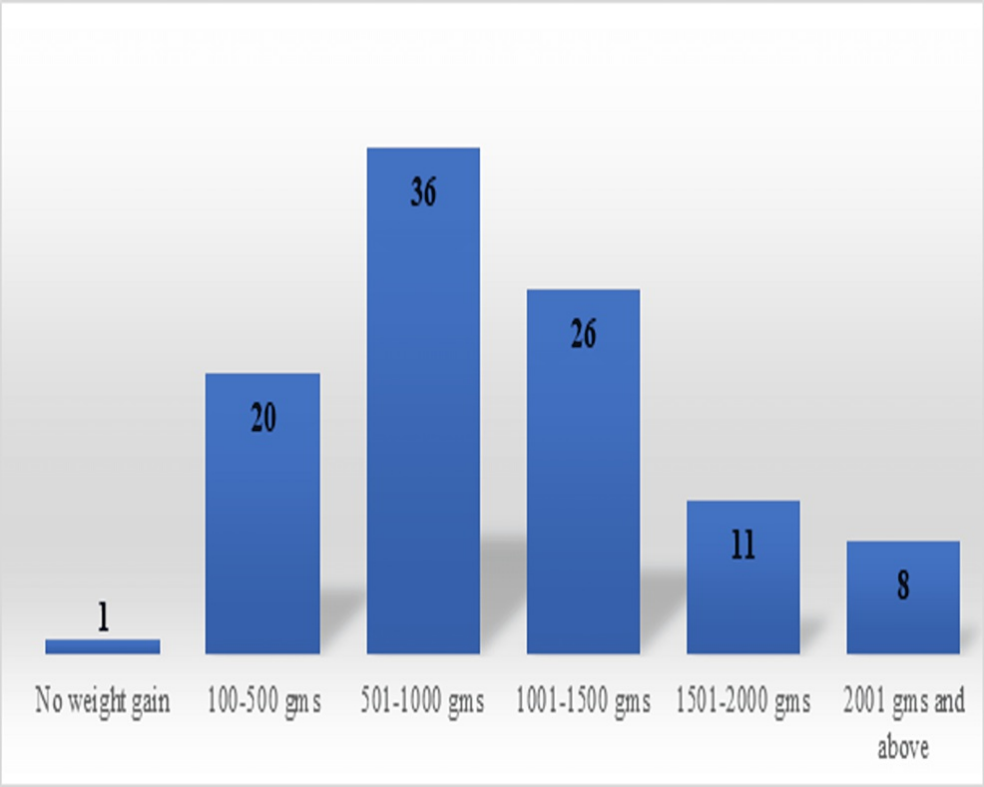
Weight gain in grams among children who completed treatment

Weight gain improvement in grams was of 100-500 gm in 20 children, 501-1000 gm in 36 children, 1001-1500 gm in 26 children, 1501-2000 gm in 11 children, and more than 2000 gm in 8 children. Only one child showed no weight gain as the child was suffering from microcephaly (Figure 3).

**Figure 4:**
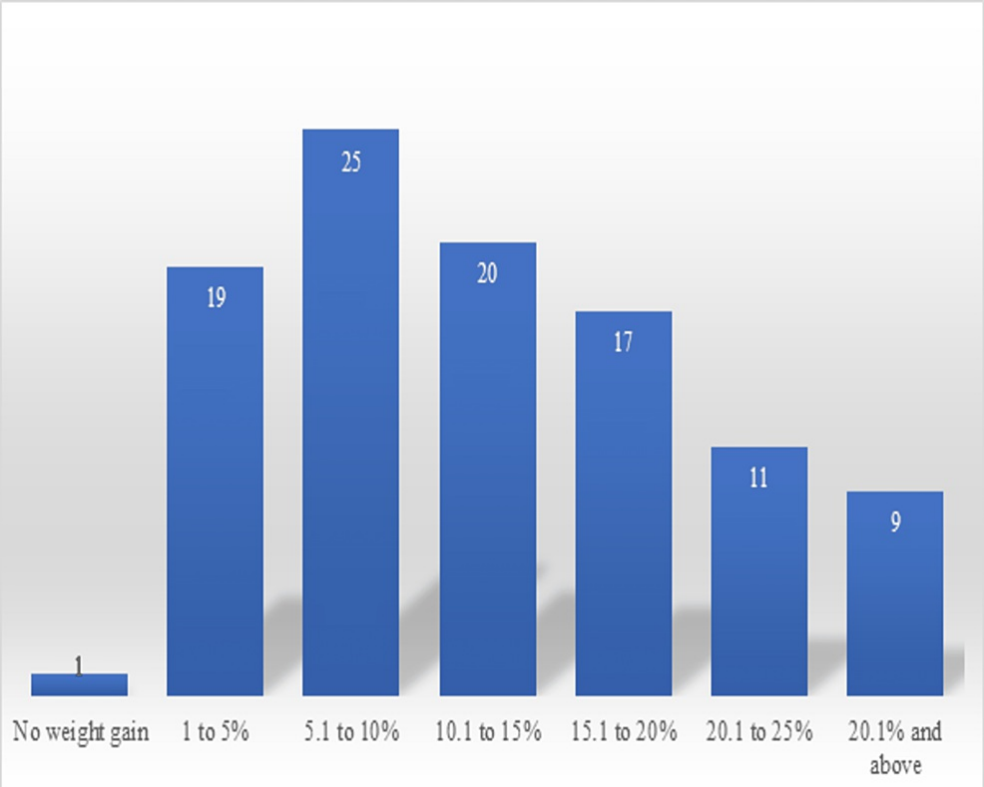
Weight gain in percentage among children who completed treatment

More than 25% weight gain was seen in nine children; 20.1%-25% weight gain was seen among 11 children, 15.1%-20% weight gain was seen in 17 children, 10.1%-15% weight gain was seen in 20 children, 5.1%-10% weight gain was seen in 25 children and 1%-5% weight gain was seen in 19 children. Only a single child showed no weight gain (Figure 4).

**Figure 5:**
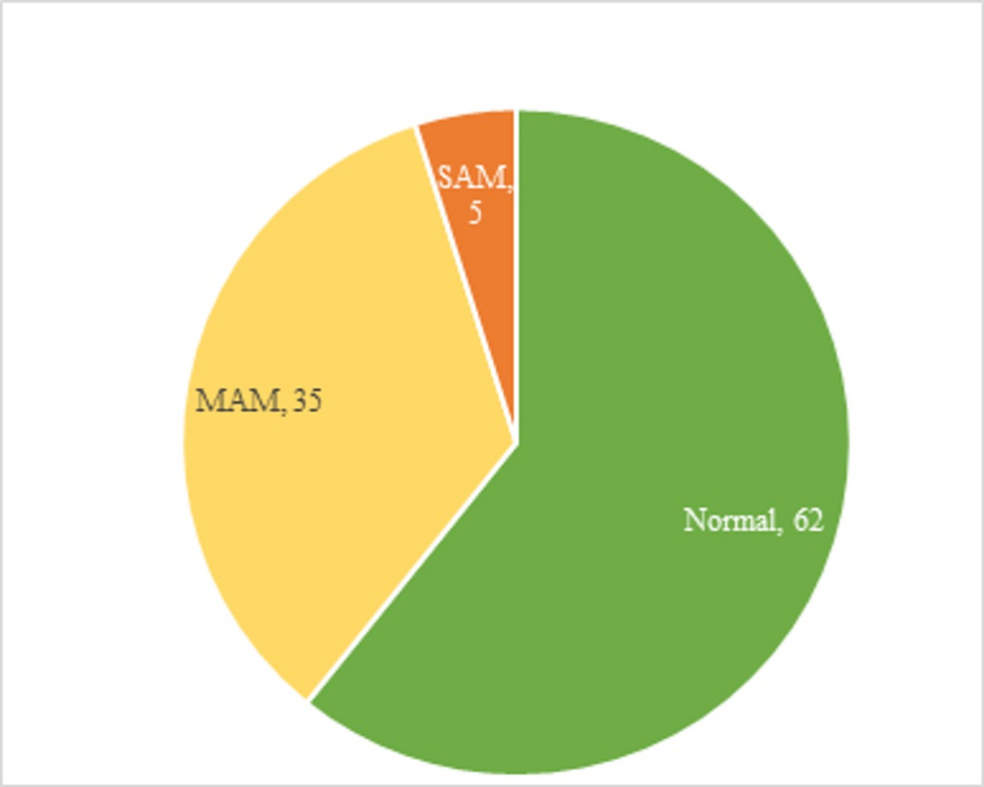
Nutritional status of children after the completion of treatment (N=102)

After the completion of treatment with three consequent follow-ups, 62 (60.79%) children had become normal in their nutritional status, 35 (34.31%) remained in moderate acute malnutrition (MAM) and 5 (4.90%) had not improved due to various reasons and remained in SAM (Figure 5). Some children showed a decline in weight gain during their respective follow-ups due to various reasons like improper diet provided at home, no proper care of child, cough, cold, fever and diarrhea episodes and non-compliance towards treatment from the parent’s side. However, parents of SAM and MAM children were counselled properly.

## Discussion

The results show that children admitted under the scheme had SAM and such children had an elevated risk of mortality as compared to non-SAM children. This study highlights the critical importance of identifying and appropriately managing children with SAM through facility-based management in the Devbhumi Dwarka district of Gujarat with a high burden of wasting and severe wasting. The piloting of the 'Bal Poshan Yojana' scheme in the district yielded positive results. It has helped 62.23% children recover from severe acute malnutrition and return to normal. The new concept of using the public-private collaboration to screen, detect and manage SAM at the facility level has proven to be an effective approach for SAM management. It has and will continue to improve adherence among recipients by enlisting the help of private practitioners and facilities, providing adequate nutrition and food, maintaining a sanitary environment, and keeping children in a designated ward. It fills treatment gaps since it prefers to see a private practitioner for SAM management.

Facility-based management of SAM children through timely feeding was overseen by a residential medical officer along with nurses and cooks for the preparation of feed and proper disbursal for the same. Daily visits by a pediatrician permitted a prompt and an effective reaction to any medical difficulty that arose, in addition to an on-site medical officer to respond to any medical need or help occurring at any time. Along with on-site medical assistance, proper counselling was given to caregivers or guardians of SAM children for maintaining proper diet post-discharge. In addition to counselling, extra attention was paid to the children's play routines in order to create a more adaptable environment for them. Children were encouraged to participate in activities that improved their cognitive talents, motor abilities, linguistic abilities and social interaction abilities.

Post-discharge follow-ups showed the eagerness of children as well as parents/guardians that indicated the effectiveness of Bal Poshan Yojana’s facility management and its unique protocol for a 14-day treatment regimen. Contrary to other findings and studies showcasing difficulty in follow-ups of cases from NRCs or CMTCs, Bal Poshan Yojana seemingly found it easier to follow up on cases as the protocol had separate financial disbursement for follow-ups that enabled the practitioners as well as parents/guardians to come forward and fulfill the required follow-ups [16].

Our scheme shows that medically complicated SAM cases can be effectively managed under the Bal Poshan Yojana. The novel scheme through PPP has the potential for providing greater coverage for facility-based complicated SAM management in response to limited capacity of NRCs and reducing mortality risk in the vulnerable group. Bal Poshan Yojana is, to our knowledge, India's first facility-based management program of severe acute malnutrition with a public-private partnership paradigm. This scheme offers a unique opportunity to improve the nutritional status of under-five age group children with SAM. It might be replicated in districts or states with a high SAM prevalence.

## Conclusions

This study is one of its kind as it highlights a unique PPP approach to combat malnutrition. Appropriate management of SAM children with medical complications by on-site specialists and focused feeding practices and counselling of parents are an effective long-lasting model of care for facility-based SAM management. Our scheme demonstrates how public-private collaboration can manage SAM cases with complex medical conditions. The findings suggest the impact of facility-based SAM management at Bal Poshan Centres in the Khambhaliya block of Devbhumi Dwarka district. We find that the management of SAM cases with medical complications at Bal Poshan Centres, along with dietary management assistance, is a successful paradigm for ensuring the proper inpatient management of SAM children with medical complications.
